# An Anti-Jamming Method against Two-Dimensional Deception Jamming by Spatial Location Feature Recognition

**DOI:** 10.3390/s21227702

**Published:** 2021-11-19

**Authors:** Zhidong Liu, Qun Zhang, Kaiming Li

**Affiliations:** 1 The Institute of Information and Navigation, Air Force Engineering University, Xi’an 710077, China; afeuzq@163.com (Q.Z.); likaiming1982@163.com (K.L.); 2 The Collaborative Innovation Center of Information Sensing and Understanding, Xi’an 710077, China; 3 The Key Laboratory for Information Science of Electromagnetic Waves, Fudan University, Shanghai 200433, China

**Keywords:** interrupted sampling repeater jamming (ISRJ), inverse synthetic aperture radar (ISAR), twinning waveform, deception jamming

## Abstract

Interrupted sampling repeater jamming (ISRJ) is an effective method for implementing deception jamming on chirp radars. By means of frequency-shifting jamming processing of the target echo signal and pulse compression during image processing, a group of false targets will appear in different spatial locations around the true target. Extracting the features of these false targets is complex and limited to existing countering methods against ISRJ. This paper proposes an anti-jamming method to identify the spatial location characteristics of two-dimensional deception false targets. By adjusting the parameters of the radar transmitted signal, the method simultaneously transmits the anti-jamming signal and carries out false target identification and elimination in the range and azimuth dimensions. Eventually, the optimal signal parameter design of the anti-jamming signal is obtained by comparing different anti-jamming strategies in the range dimension. The validity of the proposed method is proved by deducing the mathematical model between the spatial distribution characteristics of the false targets and the radar transmitted signal parameters and demonstrated by simulations.

## 1. Introduction

Inverse synthetic aperture radar (ISAR) can obtain the two-dimensional (2D) spatial position distribution of the target scattering centers by the range and azimuth compression of the echo signal [1,2,3]. Among the deception jamming technologies against ISAR, interrupted sampling repeater jamming (ISRJ) is a mature jamming method. It has a fast response time and produces realistic false targets with flexible and controllable locations [4,5,6]. Therefore, there has been extensive jamming suppression research on ISRJ.

The large class of methods for countering ISRJ can be summarized as filtering methods. A band-pass filter is designed based on a time-frequency analysis in [7] and can automatically extract non-jamming signals and eliminate false targets by constructing an energy distribution function. In [8], a method is proposed to convert the signal classification problem into a time classification problem by using a superposition bidirectional gate recursive cell network. It can accurately extract non-jamming signals and has better ISRJ suppression performance. In [9], a jamming suppression method based on the entropy function of a singular spectrum is proposed for use with a low signal-to-noise ratio. Through the entropy-based threshold detection of the echo signal, band-pass filtering and jamming suppression are realized. In [10], a time-frequency analysis of the jamming signals under three different ISRJ strategies is conducted. Different filtering methods are used based on different jamming strategies. However, the existing filtering methods achieve jamming suppression through complex signal separation algorithms, requiring the offline or online processing of the received mixed signals, and the design of relevant filters based on prior knowledge. Thus, the application scenarios are limited. Meanwhile, the filtering method processes the mixed signals at the radar receiving end, whereas at the radar transmitting end, the signal waveform can also be designed to realize jamming suppression [11]. In [12], a sensitive Doppler sparse waveform is designed based on the fuzzy function to suppress false targets by destroying the output of the jamming signal. A method for constructing a sparse target model based on Bayesian compressed sensing is proposed in [13], which only extracts discrete signals without jamming and then optimizes the target echo model to achieve jamming suppression. In [14], a method is proposed to suppress ISRJ by jointly designing the radar waveform and mismatch filter. In [12,13,14], the transmitting waveform is actively designed at the radar transmitting end, and the sparsity of targets at the spatial position is utilized to proactively avoid the output of the jamming signal in imaging processing and achieve jamming suppression. Based on the above research results, the algorithm proposed in this paper combines waveform design at the transmitter and imaging results at the receiver to identify and eliminate false targets.

ISRJ technology is essentially a type of frequency-shifting jamming against chirp ISAR [15,16]. In [17], the spatial location characteristics of false targets and the real target are analyzed, and a method to identify the false target by adjusting the radar transmission signal bandwidth is proposed. However, the range resolution declines with a change in the bandwidth of linear frequency modulation (LFM) and can only counter the false targets generated in the range direction. Traditional ISRJ only samples the target signal interruptedly in the fast time domain [18,19]. In [20,21,22], a group of false targets in two dimensions (2D) are generated by interrupted sampling in the fast and slow time domains. However, existing anti-jamming technology is unable to effectively identify false targets generated in the azimuth dimension [23,24,25]. Therefore, this paper proposes a method to actively adjust the radar signal parameters to counter the deception jamming of 2D ISRJ by studying the spatial position characteristics of the 2D false targets.

The traditional IRSJ method makes use of the relationship between the spatial positions of the false targets and the parameters of the sampling function, including the duty ratio and sampling frequency, to flexibly adjust the number and spatial positions of the false targets [26,27]. From the perspective of electronic countermeasures, if the relationship between the radar transmitted signal parameters and the spatial position of the false targets can be established, the false targets can be identified and eliminated by actively changing their spatial position distribution.

The proposed anti-jamming method needs to design two radar transmitted signals: the original radar transmitted signal and an anti-jamming signal with different signal parameters. These two signal channels are processed by matching filter imaging, respectively, and the obtained results are stored in the range and azimuth units. Then, the imaging results of the two signals are compared and judged. The false targets move within the range resolution and azimuth resolution units, while the true target stays within the same range and azimuth units, thus realizing the spatial position recognition of the true and false targets. This method does not require complicated signal analysis and processing, and false targets can be directly identified using the imaging results, which is applicable to many scenarios.

The structure of this paper is as follows. The signal model is established and the mechanism of 2D ISRJ is presented in Section 2. Section 3 presents a 2D deception jamming countermeasures analysis and the process of the proposed jamming suppression method is presented in detail. In Section 4, anti-jamming simulations are presented in different dimensions and the validity of the proposed method is proved. Finally, conclusions are drawn in Section 5.

## 2. Signal Model

**Figure 1:**
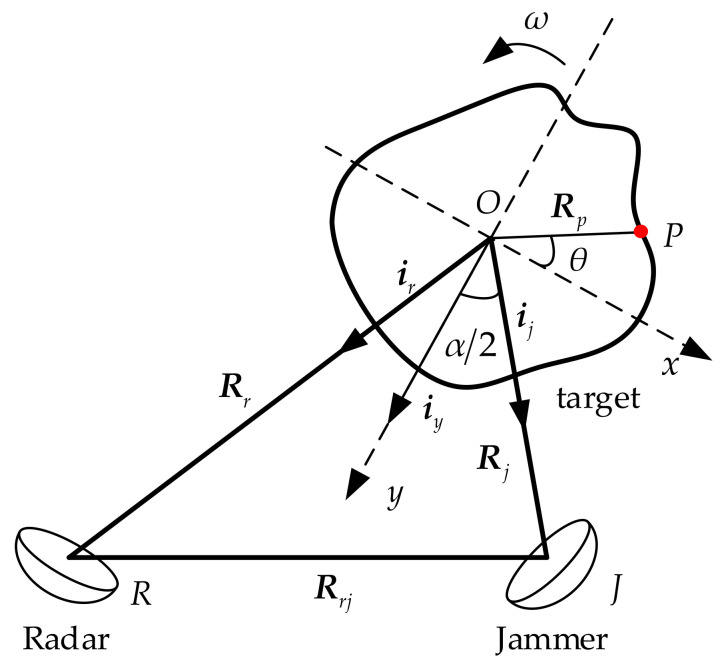
Geometric positions of ISAR, jammer and target.

Without loss of generality, the real motion of a target should include translational and rotating parts. However, because the translational component does not contribute to radar azimuth imaging, the target motion model is usually equivalent to the rotating motion model through translational compensation in the ISAR imaging process [28,29,30,31]. Based on the above assumption, the spatial geometric positions of the radar, jammer, and detected target are shown in Figure 1. The rotation center of the target is point *O*. The distance vectors from *O* to the radar and jammer are $R_{r}$ and $R_{j}$, respectively, and $R_{rj}$ is the distance vector between the radar and jammer. The reference coordinate system xoy is established by defining the bisector of the included angle α between the radar and the jammer as the y-axis. Taking point *P* on the target as an example, the position vector with respect to *O* is $R_{p}$. The initial angle between $R_{p}$ and the x-axis is θ. The rotational angular velocity of the target is ω.

The distance history of the radar transmitted signal that returns to the radar receiver after being processed by the jammer is as follows:

(1) $$\begin{array}{l} \left\|R\left(t\right)\right\|=\left\|R_{p}\left(t\right)+R_{r}\right\|+\left\|R_{p}\left(t\right)+R_{j}\right\|+\left\|R_{rj}\left(t\right)\right\|\approx \left\|R_{r}\right\|+\left\|R_{j}\right\|+\left\|R_{rj}\right\|+R_{p}\left(t\right)\cdot \left(i_{r}+i_{j}\right)\\ =\left\|R_{r}\right\|+\left\|R_{j}\right\|+\left\|R_{rj}\right\|+2R_{p}\left(t\right)\cdot i_{y}\cos\left(\alpha /2\right) \end{array}$$

When Δt is small, the distance history at time t+Δt can be expressed as follows:

(2) $$\left\|R\left(t+\Delta t\right)\right\|=\left\|R_{r}\right\|+\left\|R_{j}\right\|+\left\|R_{rj}\right\|+2\left(R_{p}\left(t\right)+\omega \Delta t\times R_{p}\left(t\right)\right)\cdot i_{y}\cos\left(\alpha /2\right)$$

If the signal waveform transmitted by radar is an LFM signal, it can be expressed as follows:

(3) $$S\left(\hat{t},t_{m}\right)=\mathrm{rect}\left(\hat{t}/T_{p}\right)\exp\left(2j\pi \left(f_{0}t+\frac{1}{2}K{\hat{t}}^{2}\right)\right)$$

where $f_{0}$ is the carrier frequency, K is the chirp rate, $\hat{t}$ is the fast time, $T_{p}$ is the pulse width, $t_{m}=mT_{d}$ is the slow time, $T_{d}$ is the pulse repetition period, m is the pulse sequence number which is in the range of 0≤m<M, and M is the total number of pulses during the entire ISAR imaging period. The total observation time is $T_{M}=MT_{d}$, $t=t_{m}+\hat{t}$ is the full time. The signal bandwidth is $B=KT_{p}$. $rect\left(\hat{t}/T_{p}\right)$ yields 1 when $\left|\hat{t}/T_{p}\right|<0.5$ and 0 otherwise.

Therefore, the Doppler frequency shift at time *t* is found as follows:

(4) $$\begin{array}{l} f_{d}=\frac{f_{0}}{c}\underset{\Delta t\to 0}{\lim}\frac{\left\|R\left(t+\Delta t\right)\right\|-\left\|R\left(t\right)\right\|}{\Delta t}=\frac{f_{0}}{c}\underset{\Delta t\to 0}{\lim}\frac{2\left(\omega \Delta t\times R_{p}\left(t\right)\right)\cdot i_{y}\cos\left(\alpha /2\right)}{\Delta t}\\ =\frac{2f_{0}}{c}\left(\omega \times R_{p}\left(t\right)\right)\cdot i_{y}\cos\left(\alpha /2\right)=\frac{2f_{0}\omega \cdot \cos\left(\alpha /2\right)y_{p}}{c} \end{array}$$

The distance of point *P* along the y-axis can be obtained using the following expression:

(5) $$y_{p}=\frac{cf_{d}}{2f_{0}\omega \cos\left(\alpha /2\right)}$$

Similarly, the distance of point *P* along the x-axis can be obtained using the following expression:

(6) $$x_{p}=\frac{c\hat{f}}{2K\cos\left(\alpha /2\right)}$$

where $\hat{f}$ is the frequency in the fast time domain.

**Figure 2:**
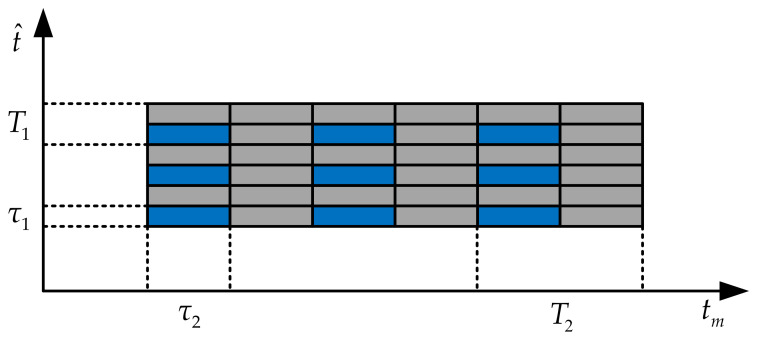
Schematic diagram of interrupted sampling in fast and slow time domains.

In Figure 2, the blue blocks represent the signal after the interrupted sampling in the fast and slow time domains, and the grey blocks represent the signals that are not sampled. The pulse width of the interrupted sampling in the fast time domain is $\tau_{1}$, and the sampling period is $T_{1}$. Similarly, the pulse width of the interrupted sampling in the slow time domain is $\tau_{2}$, and the sampling period is $T_{2}$.

The sampling functions in the fast and slow time domains are shown as follows:

(7) $$p_{1}\left(\hat{t}\right)=\mathrm{rect}\left(\hat{t}/\tau_{1}\right)*\sum_{n_{1}=-\infty }^{+\infty }\delta \left(\hat{t}-n_{1}T_{1}\right)$$

(8) $$p_{2}\left(t_{m}\right)=\mathrm{rect}\left(t_{m}/\tau_{2}\right)*\sum_{n_{2}=-\infty }^{+\infty }\delta \left(t_{m}-n_{2}T_{2}\right)$$

Based on the properties of the interrupted sampling function [31], the pulse width of radar signal $T_{p}$ must be much larger than the pulse width of the sampling period $\tau_{1}$ and sampling period $T_{1}$. Similarly, in the slow domain, the pulse repetition interval of the radar signal is less than $\tau_{2}$ and $T_{2}$. The statements are equivalent to the following two expressions:

(9) $$\tau_{1}{,T}_{1}\leq T_{p}$$

(10) $$\tau_{2}{,T}_{2}\geq T_{PRI}$$

The jamming signal $S_{r}\left(t\right)$ received by the radar receiver after interrupted sampling in the fast and slow time domains by the jammer can be expressed as follows:

(11) $$S_{r}\left(t\right)=\sigma_{Q}p_{1}\left(\hat{t}\right)p_{2}\left(t_{m}\right)\cdot S\left(t-t_{s}-t_{d}\right)$$

where $\sigma_{Q}$ is the electromagnetic scattering coefficient of the target, $t_{d}$ is the jamming processing delay, which is constant, and $t_{s}$ is the echo delay of the target.

The echo delay of reference signal $t_{ref}$ is defined as $t_{ref}=\left(\left\|R_{r}\right\|+\left\|R_{j}\right\|+\left\|R_{rj}\right\|\right)/c$. The received jamming signal is deciphered and then the fast time Fourier transform is applied to the jamming signal to obtain the following results:

(12) $$\begin{array}{l} S_{r}\left(\hat{f},t_{m}\right)=\sum_{n_{1}=-\infty }^{\infty }p_{2}\left(t_{m}\right)\cdot \sigma_{Q}\gamma_{1}T_{p}\mathrm{sinc}\left(n_{1}\gamma_{1}\right)\cdot \mathrm{sinc}\left(\left(\hat{f}-n_{1}f_{1}-K\left(t_{ref}-t_{s}-t_{d}\right)\right)T_{p}\right)\\ \cdot \exp\left(j2{\pi f}_{c}\left(t_{ref}-t_{s}-t_{d}\right)\right) \end{array}$$

where $\gamma_{1}$ is the duty ratio of the fast time sampling function. By analyzing the sinc function corresponding to the fast time frequency, it can be found that the interval of each false target in the fast frequency domain is $\Delta \hat{f}=f_{1}$. The scaling is realized by using the relation between the fast time frequency and range distance in (5).

The spatial distance between adjacent false targets can be obtained as follows:

(13) $$\Delta x_{p}=\frac{c\Delta \hat{f}}{2K\cos\left(\alpha /2\right)}=\frac{cf_{1}}{2K\cos\left(\alpha /2\right)}$$

After the range focusing is completed, the focus of the azimuth is analyzed. Here, $S_{r}\left(\hat{f},t_{m}\right)$ does not explicitly contain the slow time domain variable $t_{m}$. In contrast, $t_{s}$ contains $t_{m}$ in the following formula:

(14) $$t_{s}=\left\|R\left(t+t_{m}\right)\right\|/c=\left(\left\|R_{r}\right\|+\left\|R_{j}\right\|+\left\|R_{rj}\right\|+2\left(R_{p}\left(t\right)+\omega t_{m}\times R_{p}\left(t\right)\right)\cdot i_{y}\cos\left(\alpha /2\right)\right)/c$$

The time difference between the target echo delay and reference echo delay is as follows:

(15) $$t_{ref}-t_{s}\left(t_{m}\right)=-2\left(R_{p}\left(t\right)+\omega t_{m}\times R_{p}\left(t\right)\right)\cdot i_{y}\cos\left(\alpha /2\right)/c=-2\left(y_{p}+\omega t_{m}y_{p}\right)\cos\left(\alpha /2\right)/c$$

Substituting the result of (15) into (12), we obtain the following expression:

(16) $$\begin{array}{l} S_{r}\left(\hat{f},t_{m}\right)=\sum_{n_{1}=-\infty }^{\infty }p_{2}\left(t_{m}\right)\cdot \sigma_{Q}\gamma_{1}T_{p}\mathrm{sinc}\left(n_{1}\gamma_{1}\right)\cdot \mathrm{sinc}\left(\left(\hat{f}-n_{1}f_{1}-K\left(t_{ref}-t_{s}-t_{d}\right)\right)T_{p}\right)\\ \cdot \exp\left(j2\pi f_{c}\left(-2\left(y_{p}+\omega t_{m}y_{p}\right)\cos\left(\alpha /2\right)/c-t_{d}\right)\right) \end{array}$$

The following results are obtained by applying the Fourier transform of the slow time to (16) and ignoring the influence of $t_{m}$ on the range focusing:

(17) $$\begin{array}{l} S_{r}\left(\hat{f},f_{m}\right)=\sum_{n_{1}=-\infty }^{\infty }\sum_{n_{2}=-\infty }^{\infty }\sigma_{Q}\gamma_{1}\gamma_{2}T_{p}\mathrm{sinc}\left(n_{1}\gamma_{1}\right)\mathrm{sinc}\left(n_{2}\gamma_{2}\right)\cdot \mathrm{sinc}\left(\left(\hat{f}-n_{1}f_{1}-K\left(t_{ref}-t_{s}-t_{d}\right)\right)T_{p}\right)\\ \cdot \mathrm{sinc}\left(MT_{prf}\left(f_{m}-n_{2}f_{2}+2f_{c}y_{p}\omega \cos\left(\alpha /2\right)/c\right)\right) \end{array}$$

where *M* is the number of pulse strings and $\gamma_{2}$ is the duty ratio of the slow time sampling function. By analyzing the sinc function corresponding to the slow time frequency, it can be found that the interval of each false target in the slow time frequency domain is $\Delta f_{m}=f_{2}$. Based on Equation (5), the distance between adjacent false targets on the y-axis can be obtained as follows:

(18) $$\Delta y_{p}=\frac{c\Delta f_{m}}{2f_{c}\omega \cos\left(\alpha /2\right)}=\frac{cf_{2}}{2f_{c}\omega \cos\left(\alpha /2\right)}$$

Through the above analysis, the spatial position distribution of the 2D spatial false target generated by the 2D ISRJ can be obtained. The distance of the false targets in the range dimension $\Delta x_{p}$ is determined by the sampling frequency $f_{1}$ of the interrupted sampling function in the fast time domain, chirp rate, and bistatic angle α. The distance of the false targets in the azimuth dimension $\Delta y_{p}$ is determined by the sampling frequency $f_{2}$ of the interrupted sampling function in the slow time domain, the target’s equivalent angular velocity of rotation ω, the carrier frequency of the radar transmitted signal $f_{c}$, and the bistatic angle α.

## 3. 2D Deception Jamming Countermeasures Analysis

For the jammer, the spatial position of the false target can be changed by dynamically adjusting the relevant parameters of the interrupted sampling function and the spatial position relationship between the jammer and the target, including the distance and angle. The specific parameters include the sampling frequency, duty ratio, target’s equivalent angular velocity of rotation, and the bistatic angle. There has been much research on how to dynamically and effectively adjust the spatial positions of the 2D false targets by the jammer.

In this paper, from the perspective of jamming countermeasures, it is found that when the jammer parameters remain unchanged within a radar transmitting and receiving cycle, based on the characteristics of ISRJ technology, the radar signal parameters are actively changed to identify the positions of the false targets. According to the signal model analysis in Section 2, for ISAR, the pulse width $T_{p}$, bandwidth B, and carrier frequency of the radar transmitted signal $f_{c}$ can be adjusted to change the spatial positions of the false targets. The range and azimuth dimensions are discussed below, respectively.

### 3.1. False-Target Recognition in Range Dimension

To identify the false targets in the range dimension, the pulse width and bandwidth of the transmitting radar waveform can be actively changed without changing the spatial position of the radar system. The false targets can be identified if the change in the range dimension is greater than the resolution of the range dimension. Three different anti-jamming strategy scenarios are discussed below. It is assumed that the pulse width and bandwidth are independent of each other in Section 3.1.1 and Section 3.1.2, while in Section 3.1.3 the product of the pulse width and bandwidth remains the same for the radar transmitted signal.

#### 3.1.1. Identification by Only Changing Bandwidth

The radar range resolution is ΔR=c/2B, and the range resolution is only related to the bandwidth. After changing the bandwidth by ΔB, the spatial distance of adjacent false targets in the range dimension is as follows:

(19) $$d^{\prime }=\frac{cf_{1}T_{p}}{2\cos\left(\alpha /2\right)\left(B+\Delta B\right)}$$

The change in the spatial distance of adjacent false targets in the range dimension can be expressed as follows:

(20) $$\Delta d=\frac{cf_{1}T_{p}}{2\cos\left(\alpha /2\right)\left(B+\Delta B\right)}-\frac{cf_{1}T_{p}}{2\cos\left(\alpha /2\right)B}=-d\cdot \frac{1}{1+B/\Delta B}$$

Based on the relationship that Δd>ΔR, the maximum bandwidth change is shown below:

(21) $$\Delta B<B\left(\frac{-1}{f_{1}T_{p}+1}\right)$$

Furthermore, the range of bandwidth variation can be solved as follows:

(22) $$B^{\prime }<B\left(1-\frac{1}{1+f_{1}T_{p}}\right)$$

To realize false-target recognition in the range dimension, the bandwidth $B^{\prime }$ of the anti-jamming signal should be less than $B\left(1-\frac{1}{1+f_{1}T_{p}}\right)$, and the pulse width can be obtained directly by the ISAR radar system, but sampling frequency $f_{1}$ is determined by the jammer and must be estimated.

#### 3.1.2. Identification by Only Changing Pulse Width

After changing the pulse width by $\Delta T_{p}$, the change in the spatial distance of adjacent false targets in the range dimension can be expressed as follows:

(23) $$\Delta d=\frac{cf_{1}\left(T_{p}+\Delta T_{p}\right)}{2B}-\frac{cf_{1}T_{p}}{2B}=cf_{1}\Delta T_{p}/2B$$

Based on the relationship Δd>ΔR, the minimum pulse width change is as follows:

(24) $$\Delta T_{p}>1/f_{1}$$

Furthermore, the range of pulse width variation can be solved as follows:

(25) $$T_{p}^{\prime }>T_{p}\left(1+\frac{1}{f_{1}T_{p}}\right)$$

To realize the false-target recognition in the range dimension, the pulse width $T_{p}^{\prime }$ of the anti-jamming signal should be greater than $T_{p}\left(1+\frac{1}{f_{1}T_{p}}\right)$. The pulse width can be obtained directly by the ISAR radar system, but sampling frequency $f_{1}$ is decided by the jammer and must be estimated.

#### 3.1.3. Identification by Changing Pulse Width and Bandwidth Synchronously

In this subsection, the pulse width and bandwidth are changed synchronously. Because the product of the pulse width and bandwidth is unchanged, the relationship between the change in the pulse width and the change in the bandwidth can be deduced as follows:

(26) $$\Delta T_{p}=\frac{-\Delta B}{\Delta B+B}T_{p}$$

After changing the bandwidth by ΔB and the pulse width by $\Delta T_{p}$, the spatial distance of adjacent false targets in the range dimension is as follows:

(27) $$d^{\prime }=\frac{cf_{1}\left(T_{p}+\Delta T_{p}\right)}{2\left(B+\Delta B\right)}=\frac{cf_{1}T_{p}B}{2{\left(B+\Delta B\right)}^{2}}$$

The change in the spatial distance of adjacent false targets in the range dimension can be expressed as follows:

(28) $$\Delta d=\frac{cf_{1}T_{p}B}{2{\left(B+\Delta B\right)}^{2}}-\frac{cf_{1}T_{p}}{2B}=\left(cf_{1}T_{p}/2\right)\cdot \left(\frac{-2\Delta B\cdot B-\Delta B^{2}}{B{\left(B+\Delta B\right)}^{2}}\right)$$

Furthermore, the range of bandwidth variation can be solved as follows:

(29) $${\left(\frac{\Delta B}{B}\right)}^{2}+2\frac{\Delta B}{B}+\frac{1}{f_{1}T_{p}+1}=0$$

By solving Equation (29), the change in the bandwidth can be expressed as follows:

(30) $$\Delta B=B\cdot \left(-1\pm \sqrt{\frac{f_{1}T_{p}}{f_{1}T_{p}+1}}\right)$$

To reduce the burden of the radar system and achieve a better imaging effect, a solution with less bandwidth variation is selected, as shown in Equation (31).

(31) $$\Delta B=B\left(-1+\sqrt{\frac{f_{1}T_{p}}{f_{1}T_{p}+1}}\right)$$

Meanwhile, the change in the pulse width can be expressed as follows:

(32) $$\Delta T_{p}=\left(-1+\sqrt{\frac{f_{1}T_{p}+1}{f_{1}T_{p}}}\right)T_{p}$$

The radar resolution is mainly determined by the bandwidth of the radar transmitted signal. To minimize the loss of radar detection performance, the variations in the radar range resolution with the sampling frequency under the above, three different jamming strategies are studied.

**Figure 3:**
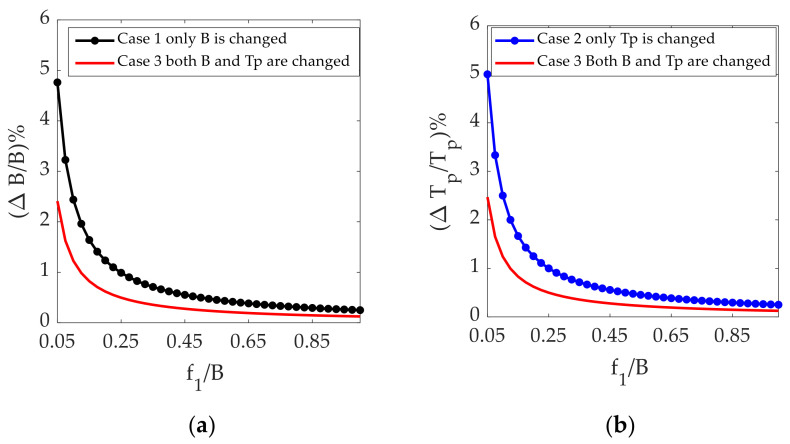
Analysis results of different jamming strategies: (**a**) ratio of bandwidth variation to bandwidth of signal vs. sampling frequency shift relative to bandwidth; (**b**) ratio of pulse width variation to pulse width of signal vs. sampling frequency shift relative to bandwidth.

In Figure 3a, the black dotted curve represents the ratio of the bandwidth variation to the original bandwidth of the signal at different sampling frequencies in case 1, where only *B* is changed. The red curve represents the ratio of the bandwidth change to the original bandwidth of the signal at different sampling frequencies in case 3, where both *B* and *T_p_* are changed.

In Figure 3b, the blue dotted curve represents the ratio of the pulse width change to the original pulse width of the signal at different sampling frequencies in case 2, where only *T_p_* is changed. The red curve represents the ratio of the pulse width change to the original pulse width of the signal at different sampling frequencies in case 3, where both *B* and *T_p_* are changed.

It can be concluded from Figure 3a,b that the bandwidth and pulse width required in case 3 are both lower than those required in case 1, where only the pulse width is changed, and case 2, where only the bandwidth is changed, thus avoiding the degradation of the radar performance.

**Figure 4:**
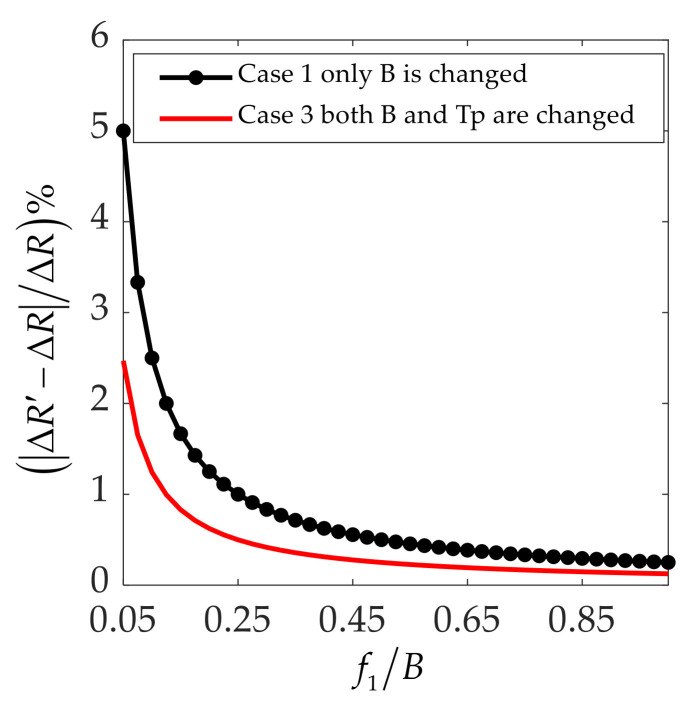
Degradation of radar range resolution vs. jammer frequency shift relative to bandwidth.

In Figure 4, the red curve is lower than the black dotted curve, which proves that the degradation of the radar range in case 3 is less than that in case 1. Based on the above analysis and discussion, it is concluded that the best anti-jamming strategy in the range dimension is to change the bandwidth and pulse width at the same time to minimize the loss of radar performance.

### 3.2. False-Target Recognition in Azimuth Dimension

To identify false targets in the azimuth dimension, the carrier frequency of the transmitting radar waveform can be actively changed without changing the spatial position of radar system. The azimuth resolution is defined as ΔR=λ/2f, where λ is the wavelength of the radar signal, and ϕ is the image accumulation angle. After changing the carrier frequency to $f_{c}^{\prime }$, the spatial distance between the adjacent false targets in the range dimension is as follows:

(33) $$d^{\prime }=\frac{cf_{2}}{2f_{c}^{\prime }\omega \cos\left(\alpha /2\right)}$$

The change in the spatial distance of adjacent false targets in the azimuth dimension can be expressed as follows:

(34) $$\Delta d=\frac{cf_{2}}{2\omega \cos\left(\alpha /2\right)}\left(\frac{1}{f_{c}^{\prime }}-\frac{1}{f_{c}}\right)$$

Based on the relationship Δd>ΔR, the maximum carrier frequency change is expressed as follows:

(35) $$f_{c}^{\prime }>f_{c}\left(1-\frac{\omega \cos\left(\alpha /2\right)}{f_{2}\phi +\omega \cos\left(\alpha /2\right)}\right)$$

where $C=\frac{\omega \cos\left(\alpha /2\right)}{f_{2}\phi }$. Furthermore, the carrier frequency range can be solved as follows:

(36) $$f_{c}^{\prime }>f_{c}/\left(1+C\right)$$

It can be found that to identify false targets in the azimuth dimension, the carrier frequency after being changed must be more than $f_{c}/\left(1+C\right)$. Based on its definition, parameter *C* is determined by the sampling frequency $f_{2}$ of the interrupted sampling function in the slow time domain, the target’s equivalent angular velocity of rotation ω, the image accumulation angle ϕ, and the bistatic angle α.

### 3.3. Spatial Location Feature Recognition Anti-Jamming Method

Based on the above analysis, this paper summarizes a jamming suppression method for 2D deception jamming by spatial location feature recognition. The key to this method is to design an anti-jamming signal $S_{2}\left(t\right)$, that is similar to the radar signal, but has a different pulse width $T_{p}^{\prime }$, bandwidth $B^{\prime }$, and carrier frequency $f_{c}^{\prime }$ which can be expressed as follows:

(37) $$S_{2}\left(t\right)=\mathrm{rect}\left(\frac{\hat{t}}{T_{p}^{\prime }}\right)\exp\left(2j\pi \left(f_{c}^{\prime }t+\frac{1}{2}\frac{B^{\prime }}{T_{p}^{\prime }}{\hat{t}}^{2}\right)\right)$$

For convenience in the later analysis and discussion, the original radar signal $S_{1}\left(t\right)$ is defined as follows:

(38) $$S_{1}\left(t\right)=\mathrm{rect}\left(\frac{\hat{t}}{T_{p}}\right)\exp\left(2j\pi \left(f_{c}t+\frac{1}{2}\frac{B}{T_{p}}{\hat{t}}^{2}\right)\right)$$

**Figure 5:**
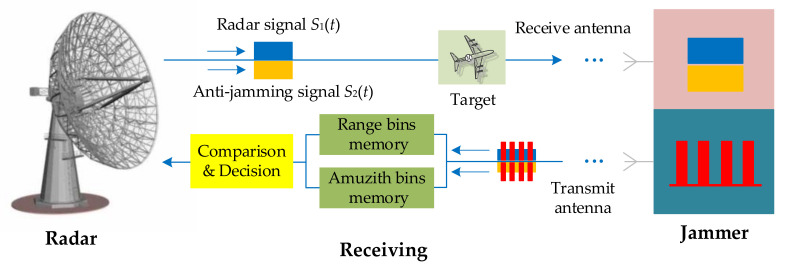
Jamming flowchart.

Figure 5 shows a schematic diagram of the proposed anti-jamming technique, which is based on traditional ISAR systems. The transmitting process includes the original radar signal $S_{1}\left(t\right)$ and the anti-jamming signal $S_{2}\left(t\right)$ which can be transmitted and received through two channels individually and synchronously without jamming. Effective signal separation can be achieved when the signal bandwidth has a different frequency range. When the bandwidths of the two signals partially overlap, the two signals need to be designed differently by combining the transmitted pulses. For example, pulse diversity technology or sub-pulse technology can be used to transmit two signals using different pulse sequences or sub-pulses, which are then separated at the receiving end of the radar to ensure that the two signals do not jam each other.

Specifically, the radar transmits the original radar signal and anti-jamming signal and stores the target space positions detected with the two signals in different range bins and azimuth bins.

**Figure 6:**
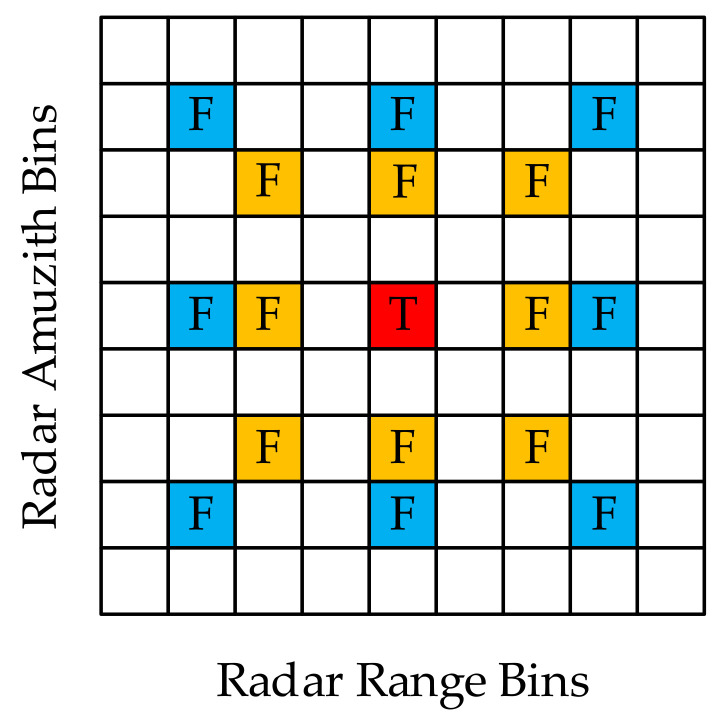
Comparison and decision, where red bin represents the true target, blue bins represent the false targets found with transmission of $S_{1}\left(t\right)$, and yellow bins represent the false targets found with transmission of $S_{2}\left(t\right)$.

In Figure 6, T represents the true target and F represents the false target. A comparison of the imaging results for the two signals shows that the true target stays in the same range and azimuth bins, while the false target appears in different range and azimuth bins and can therefore be distinguished from the true target by judging the spatial position characteristics of the targets.

**Figure 7:**
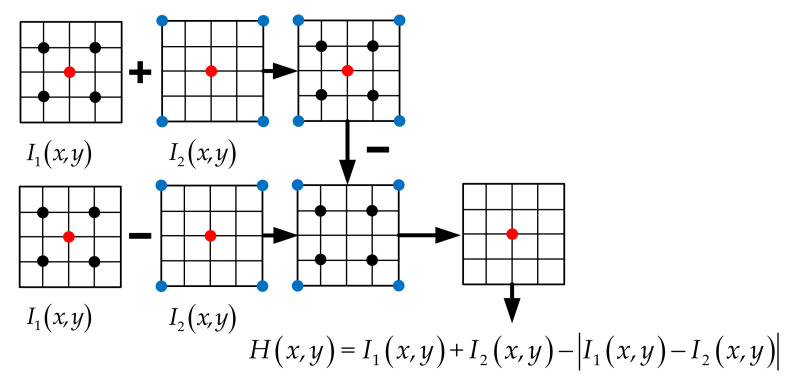
Flowchart of spatial location identification function for true and false targets.

Based on the twinning waveforms transmitted by the radar, the spatial location identification function of the true and false targets $H\left(x,y\right)$ is constructed as follows:

(39) $$H\left(x,y\right)=I_{1}\left(x,y\right)+I_{2}\left(x,y\right)-\left|I_{1}\left(x,y\right)-I_{2}\left(x,y\right)\right|$$

where $I_{1}\left(x,y\right)$ and $I_{2}\left(x,y\right)$ are the imaging results corresponding to the two signals. First, the two imaging results are summed. Secondly, the two imaging results are subtracted and the absolute value is taken, and the absolute value is subtracted from the sum result to get the final suppression result. Thus, the cancellation of false targets and the reservation of real targets can be achieved. The flowchart for the spatial location identification function of the true and false targets is shown in Figure 7. The red dots represent the real target, while the black and blue dots represent the false targets generated by two different jamming signal channels.

Because of the limitation of the imaging algorithm, a sidelobe exists in the actual imaging results, which means that the target is not fully focused within a resolution unit. Because the anti-jamming method in this paper can only make false targets move by a resolution unit, when the target is not focused on a resolution within the unit, the phenomenon of aliasing between the two imaging results will occur. Based on this analysis, two imaging results need to be preprocessed to eliminate the side lobe, ensuring the accuracy of the true and false target space position identification function. 

**Figure 8:**
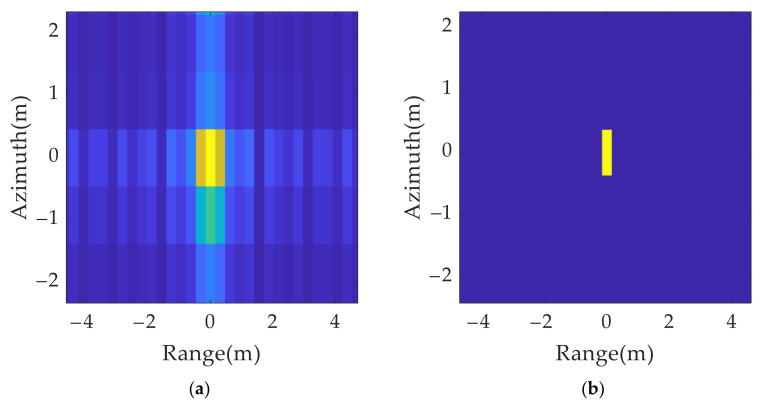
Comparison of single scattering point’s imaging results before and after side lobe elimination: (**a**) without side lobe elimination; (**b**) with side lobe elimination.

The solution is as follows: the resolution unit corresponding to the maximum scattering intensity in the imaging results is determined by a local peak search. It is retained, and the other position units are set to zero in both the range and azimuth dimensions. It can be concluded from Figure 8 that, after the sidelobe elimination, the imaging results of the point targets are focused within a resolution unit.

When the target model has multiple scattering points, even if there is no influence from imaging sidelobe, the two imaging results must be aliased between multiple points. Based on the aliasing of imaging results, the spatial location identification function cannot completely eliminate false targets; it is necessary to convert multiple scattering point models into a single scattering point model by choosing the strongest scattering points. After eliminating the false objects using spatial location identification function, the real objects are retained by spatial position mapping.

## 4. Simulations

In this section, the performance of the anti-jamming method is validated by simulations. The main simulation parameters are listed in Table 1.

### 4.1. 2D Deception Jamming Simulation

The main jamming simulation parameters are listed in Table 2. The sampling frequency $f_{1}$ in the fast domain is 7.5 MHz, and in the slow domain it is 3.13 Hz. To achieve a better deception effect, a false target distribution with smaller amplitude attenuation is often selected. Therefore, in practical jamming applications, a value of 0.5 is often selected for duty ratios $\gamma_{1}$ and $\gamma_{2}$ in the fast and slow domains, respectively, for the convenience of the implementation and simplification of design [31]. 

After 2D ISRJ, a group of false targets appeared around the real target. Based on the signal parameters in Table 1 and the jamming parameters listed in Table 2, the distance between the true target and false target can be calculated using $\Delta x_{p}=cf_{1}/2K\cos\left(\alpha /2\right)=5.63\;\left(m\right)$ and $\Delta y_{p}=cf_{2}/2f_{c}\omega \cos\left(\alpha /2\right)=2.93\;\left(m\right)$ for the range and azimuth dimension, respectively. 

**Figure 9:**
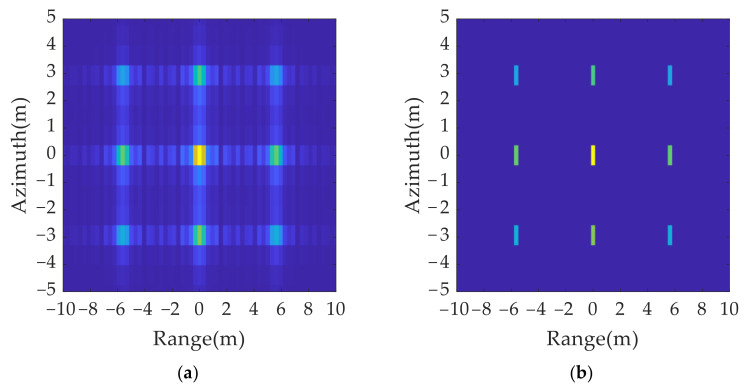
2D deception jamming results: (**a**) jamming results without eliminating sidelobe; (**b**) jamming results after eliminating sidelobe.

According to the simulation results in Figure 9, the spatial distribution of the 2D group of false targets conformed to the theoretical analysis, which verified the effectiveness of the 2D ISRJ theory. Figure 9b shows the processing result of Figure 9a after the peak search by resolution unit and sidelobe suppression.

### 4.2. Anti-Jamming Results with Single Point

According to the radar transmitter analysis in Section 3, the signal parameters, including the bandwidth, pulse width, and carrier frequency can be actively changed to determine the movement of the false target’s spatial position. In theory, the range resolution can be expressed as $\Delta R_{x}=c/2B=0.75\;\left(m\right)$ and the azimuth resolution can be expressed as $\Delta R_{y}=\lambda /2\phi =0.73\;\left(m\right)$.

Simulations based on three range dimension anti-jamming strategies were conducted. In case 1, only the bandwidth is changed to 176 MHz and the pulse width remains unchanged. In case 2, only the pulse width is changed to 1.13 µs and the bandwidth remains unchanged. In case 3, both the bandwidth and pulse width are changed, with the bandwidth changes to 187 MHz, and the pulse width changes to 1.06 µs. The main anti-jamming simulation parameters in the range domain are listed in Table 3.

**Figure 10:**
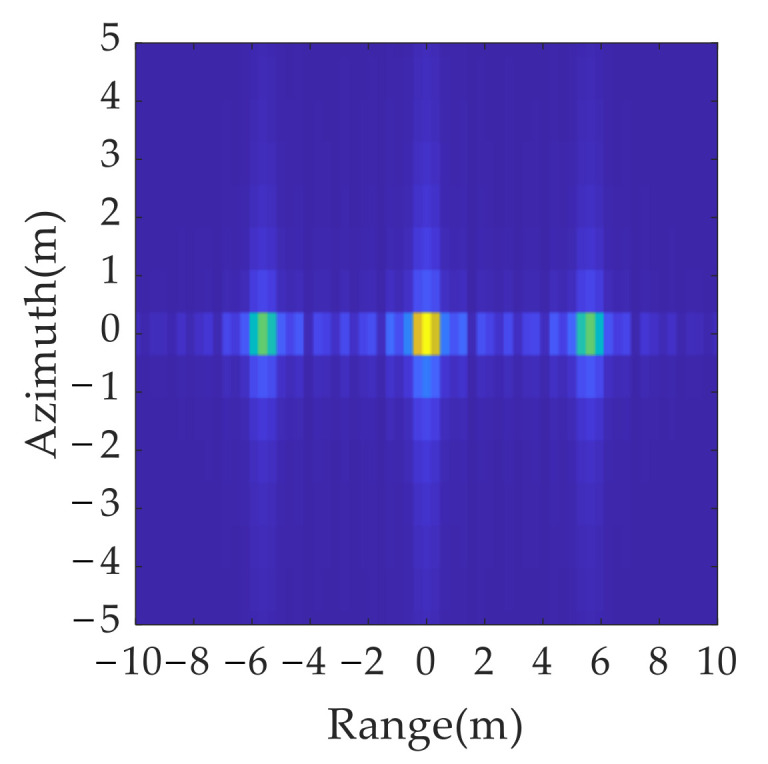
Jamming results in range dimension.

**Figure 11:**
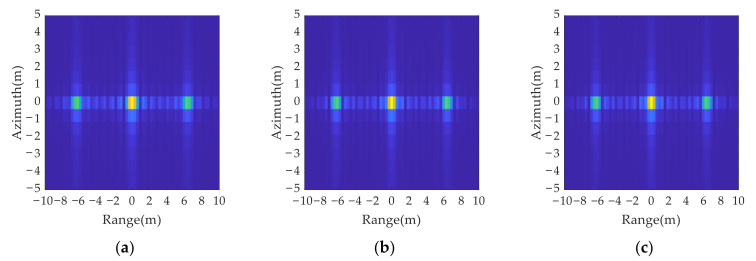
Anti-jamming results in range dimension with three different anti-jamming strategies: (**a**) case 1, where only bandwidth is changed; (**b**) case 2 where only pulse width is changed; (**c**) case 3, where both the bandwidth and pulse width are changed.

A comparison of the imaging results in Figure 10 and Figure 11 shows that all three anti-jamming strategies can determine the movement of false targets by range resolution unit, but the different strategies have different requirements for the radar system. In cases 1 and 2 only a single parameter is changed, at the cost of reducing the radar resolution or increasing the signal bandwidth. In case 3, the signal bandwidth and pulse width are changed at the same time, and the variation is smaller than those in case 1 and case 2, which has lower requirements for radar system and is easier to realize. Therefore, case 3 is chosen as the criterion for the anti-jamming parameter design in range dimension.

Based on the analysis of the spatial location feature of the false targets in the azimuth dimension, the carrier frequency of the anti-jamming signal is changed to determine the movement of false targets in the azimuth dimension according to Equation (36). The carrier frequency is changed from 8 GHz to 6.4 GHz. The main anti-jamming simulation parameters in the azimuth domain are listed in Table 4.

**Figure 12:**
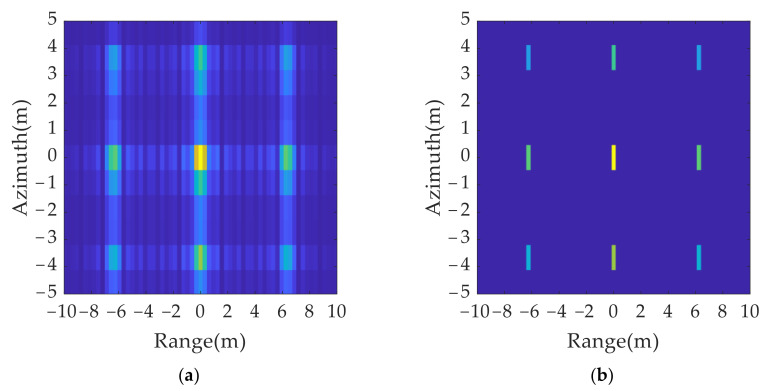
2D deception jamming results by twinning waveform: (**a**) jamming results without eliminating sidelobe; (**b**) jamming results after eliminating sidelobe.

The imaging results of the anti-jamming signals are shown in Figure 12a. Compared with Figure 9a, the positions of the false targets moved, while the positions of the real targets did not change. Figure 12b shows the results after the elimination of the sidelobe which are the same as those in Figure 9b.

**Figure 13:**
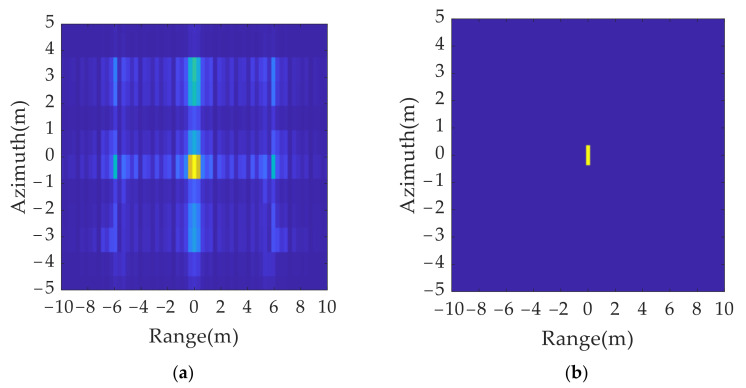
2D deception anti-jamming results: (**a**) anti-jamming results without eliminating sidelobe; (**b**) anti-jamming results after eliminating sidelobe.

According to the true and false target discriminant function proposed in this paper, a set of jamming suppression results were obtained based on Figure 9a and Figure 12a, as shown in Figure 13a. It can be found that the amplitude of the false target was greatly reduced, but because of the influence of the side lobe, there was still a residual shadow around the false target position, which affected the judgment of the real target position. The jamming suppression results after eliminating the sidelobe were obtained based on Figure 9b and Figure 12b, as shown in Figure 13b. It can be found that only real targets were retained in the inhibition results, which ensured the accuracy of the identification of real and false targets.

To evaluate the performance of the proposed anti-jamming algorithm, the entropy of images and Root Mean Square Error (RMSE) are calculated using different anti-jamming signals. The entropy is defined as follows:

(40) $$Entropy=-\sum_{m=1}^{M}\sum_{n=1}^{N}I\left(m,n\right)\ln\left[I\left(m,n\right)\right]$$

where $I\left(m,n\right)=\left|I_{0}\left(m,n\right)\right|/\sum_{m=1}^{M}\sum_{n=1}^{N}\left|I_{0}\left(m,n\right)\right|$. $I_{0}(m,n)$ is a gray value of the imaging results, located at the coordinates (m,n), and the entire imaging size is M×N. 

The RMSE between the ideal image without jamming and the reconstructed image with the proposed anti-jamming approach is defined as follows:

(41) $$\mathrm{RMSE}=\sqrt{\frac{1}{M\times N}\sum_{m=1}^{M}\sum_{n=1}^{N}{\left(\hat{I}\left(m,n\right)-\bar{I}\left(m,n\right)\right)}^{2}}$$

where $\bar{I}\left(m,n\right)$ is a gray value of the ideal imaging results without jamming, located at the coordinates (m,n). $\hat{I}\left(m,n\right)$ is a gray value of the imaging result after jamming suppression. It should be noted that all the imaging results were normalized and the value range is (0,255).

As revealed in Table 5, the entropy of the results without eliminating the sidelobe in Figure 13a is 0.4978 dB and the RMSE is 2.1302. After performing the sidelobe elimination, the entropy in Figure 13b decreased to 0.4302 dB and the RMSE decreased to 0.1684, which means that the false targets are completely eliminated, and the true target is retained.

### 4.3. Anti-Jamming Results with Multiple Points

**Figure 14:**
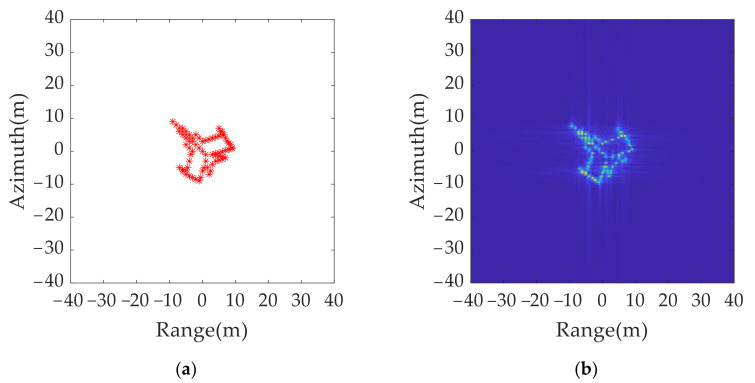
Simulated multiple points model: (**a**) aircraft model of 74 points; (**b**) ISAR imaging without jamming.

To further verify the effectiveness of the proposed algorithm, a simulated multiple points aircraft model and ISAR imaging without jamming are shown in Figure 14.

After 2D ISRJ, a group of false targets appeared around the real target. Based on the signal parameters in Table 1 and the jamming parameters in Table 6, the distance between the true target and the false target can be calculated using $\Delta x_{p}=cf_{1}/2K\cos\left(\alpha /2\right)=90\;\left(m\right)$ for the range dimension and $\Delta y_{p}=cf_{2}/2f_{c}\omega \cos\left(\alpha /2\right)=46.87\;\left(m\right)$ for the azimuth dimension. 

Simulations based on the anti-jamming strategies in case 3 were conducted. In case 3, both the bandwidth and pulse width were changed, with the bandwidth changing to 199 MHz, and the pulse width changing to 1.0042 µs. Based on the analysis of the spatial location feature of the false target in the azimuth dimension, the carrier frequency was changed from 8 GHz to 7.88 GHz. The anti-jamming simulation parameters are listed in Table 7. 

**Figure 15:**
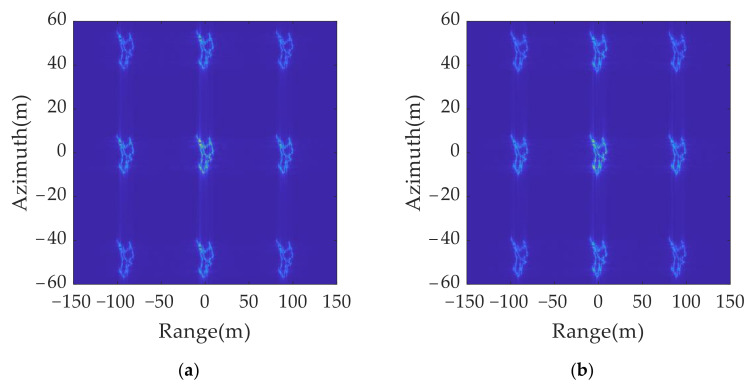
2D jamming results with different signal parameters: (**a**) original transmitted signal; (**b**) anti-jamming twinning signal.

The imaging results for the original transmitted signal and anti-jamming signals are shown in Figure 15. A comparison of Figure 15a,b shows that, although the false target moves by a resolution unit in the range and azimuth dimension, the distribution range of the multi-point false target is much larger than the resolution size. Thus, the true and false target movement cannot be directly judged by using the two imaging results.

**Figure 16:**
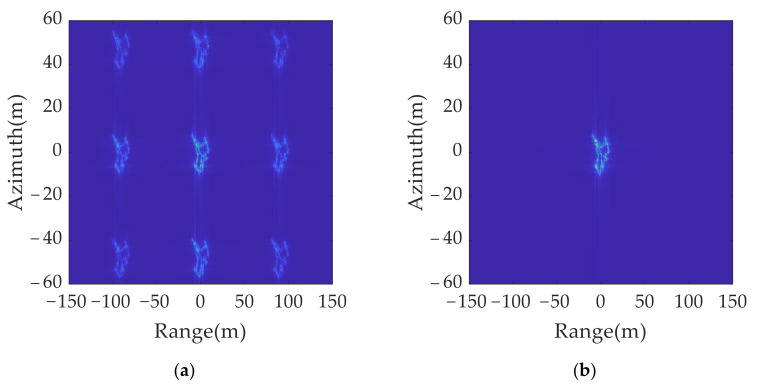
2D deception anti-jamming results: (**a**) anti-jamming results without eliminating sidelobe; (**b**) anti-jamming results after eliminating sidelobe and spatial position mapping.

A set of jamming suppression results were obtained based on Figure 15a,b using the true and false target discriminant function proposed in this paper, as shown in Figure 16a. It can be found that the amplitude of the false target was greatly reduced, but because of the influence of the side lobe, there was still a residual shadow around the false target position, which affected the judgment of the real target position. The jamming suppression results were obtained after the sidelobe was eliminated and spatial position mapping was performed, as shown in Figure 16b. Only real targets were retained in the inhibition results, which ensured the accuracy of the identification of real and false targets.

As shown in Table 8, the entropy of the original jamming results in Figure 15a is 0.7364 dB, and the RMSE is 16.6018. For the anti-jamming, the entropy of the results without eliminating the sidelobe in Figure 16a is 0.7196 dB, and the RMSE is 12.5382. After performing the sidelobe elimination and spatial position mapping, the entropy in Figure 16b decreased to 0.5434 dB and the RMSE decreased to 1.0568, which meant the false targets were completely eliminated and the true target was retained.

### 4.4. Anti-Jamming Results with Yak-42 Model Data

This subsection shows how the 2D-ISRJ algorithm was applied to measured data for a Yak-42, with a range of 35 m and an azimuth of 37 m. The signal parameters were the same as those of the single-point and multiple-point models.

**Figure 17:**
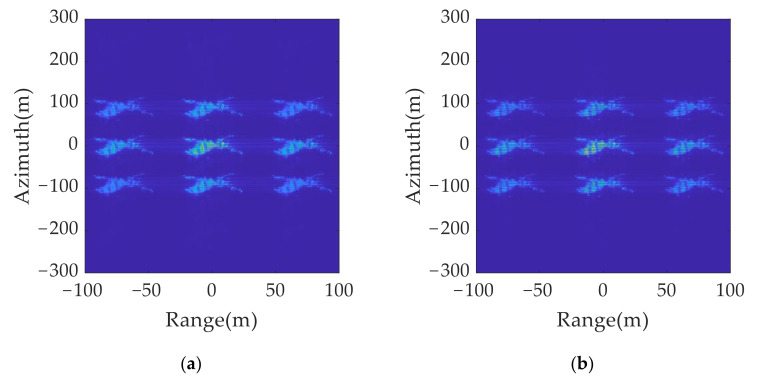
2D jamming results with different signal parameters: (**a**) original transmitted signal; (**b**) anti-jamming twinning signal.

**Figure 18:**
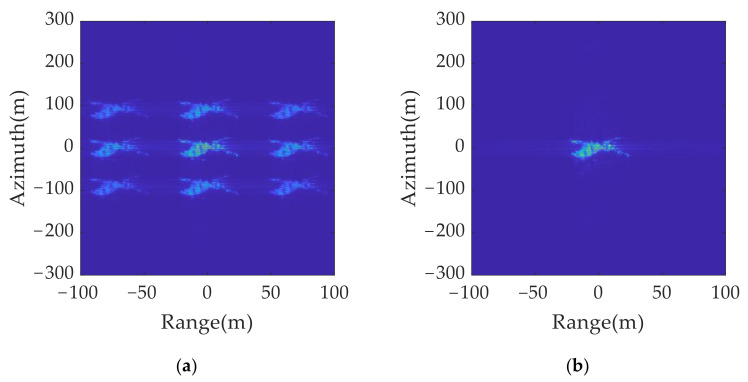
2D deception anti-jamming results: (**a**) anti-jamming results without eliminating sidelobe; (**b**) anti-jamming results after eliminating sidelobe and performing spatial position mapping.

As listed in Table 9, the entropy of the original jamming results in Figure 17a is 0.5508 dB, and the RMSE is 27.8921. For the anti-jamming, the entropy of the results without eliminating the sidelobe in Figure 18a is 0.5448 dB, and the RMSE is 20.5304. After performing the sidelobe elimination and spatial position mapping, the entropy in Figure 18b decreased to 0.4260 dB, and the RMSE decreased to 1.3951, which meant the false targets were completely eliminated and the true target was retained.

The following conclusions can be drawn based on the simulations.

(1) The relationship between the spatial location distribution of 2D false targets generated by ISRJ and the signal parameters is analyzed, and a relevant mathematical model is established.
(2) Based on the resolution, two similar twinning waveforms are designed, and the spatial position of false targets can be moved by actively adjusting the three important parameters of the transmitted signal bandwidth, pulse width, and carrier frequency, which provides a basis for comparing information to identify true and false targets.
(3) Based on the two imaging results, the true and false target discrimination function is designed, and the effects of the sidelobe and multipoint targets in the imaging on the discrimination function are discussed.
(4) In this paper, a jamming suppression method based on the spatial location features of false targets is combined with the imaging results of radar transmitter and receiver for joint design processing to avoid complex filtering and feature the extraction of signals. Furthermore, the waveform structures of the two signals is consistent, and only minor adjustment of the relevant core parameters is required, which indicated low requirements for the radar system, making it possible to quickly determine true and false targets and eliminate the false ones.

## 5. Conclusions

By analyzing a model of 2D deception false targets based on ISRJ technology, this paper proposes a new false-target recognition method based on spatial position recognition. From the perspective of signal parameter design for the radar transmitter, the original radar signal and anti-jamming signal in two simultaneous transmission channels are designed based on the mathematical model of spatial position distribution of the false targets. The target imaging results are compared and analyzed to identify and eliminate false targets. Meanwhile, the design of anti-jamming signals is based on an analysis of the radar resolution, and different jamming strategies are studied. Finally, an optimal waveform design is obtained. Compared with the existing anti-jamming methods, the proposed method avoids the filtering processing of the echo signal and more complex signal processing algorithms, and the application scenarios are more extensive. The method proposed in this paper provides a new idea for ISAR imaging countermeasures, which can be combined with additional radar performance indicators to further optimize the design of countermeasures against jamming signals in the future.

## Figures and Tables

**Table 1:** Simulation parameters.

Parameters	Numerical Value	Parameters	Numerical Value
$f_{0}\;\left(GHz\right)$	8	$\mathrm{PRF}\;\left(Hz\right)$	200
$B\;\left(MHz\right)$	200	$\omega \;\left(\mathrm{rad}\right)$	0.02
$T_{p}$ (μs)	1	$\alpha \;\left(\mathrm{rad}\right)$	0
$R_{j}\;\left(\mathrm{km}\right)$	0.5	$R_{r}\;\left(\mathrm{km}\right)$	2

**Table 2:** Jamming simulation parameters.

Parameters	Numerical Value	Parameters	Numerical Value
$f_{1}\;\left(MHz\right)$	7.5	$f_{2}\;\left(Hz\right)$	3.13
$\gamma_{1}$	0.5	$\gamma_{2}$	0.5

**Table 3:** Anti-jamming simulation parameters in range domain with three different anti-jamming strategies.

Parameters	Numerical Value in Case 1	Numerical Value in Case 2	Numerical Value in Case 3
$B^{\prime }\;\left(MHz\right)$	176	200	187
$T_{p}$ (μs)	1	1.13	1.06

**Table 4:** Anti-jamming simulation parameters in azimuth domain.

Parameters	Numerical Value	Parameters	Numerical Value
$f_{c}\;\left(GHz\right)$	8	$f_{c}^{\prime }\;\left(GHz\right)$	6.4

**Table 5:** Imaging entropies and RMSE values of two different algorithms.

	The Anti-Jamming Results without Eliminating the Sidelobe	The Anti-Jamming Results after Eliminating the Sidelobe
Entropy	0.4978 dB	0.4302 dB
RMSE	2.1302	0.1684

**Table 6:** Jamming simulation parameters.

Parameters	Numerical Value	Parameters	Numerical Value
$f_{1}\;\left(MHz\right)$	120	$f_{2}\;\left(Hz\right)$	50
$\gamma_{1}$	0.5	$\gamma_{2}$	0.5

**Table 7:** Anti-jamming simulation parameters.

Parameters	Numerical Value	Parameters	Numerical Value
$B\;\left(MHz\right)$	200	$B^{\prime }\;\left(MHz\right)$	199
$T_{p}$ (μs)	1	$T_{p}^{\prime }$ (μs)	1.0042
$f_{c}\;\left(GHz\right)$	8	$f_{c}^{\prime }\;\left(GHz\right)$	7.88

**Table 8:** Imaging entropies and RMSE values of three different algorithms.

	The 2D ISRJ Results	The Anti-Jamming Results without Eliminating Sidelobe	The Anti-Jamming Results in This Paper
Entropy	0.7364 dB	0.7196 dB	0.5434 dB
RMSE	16.6018	12.5382	1.0568

**Table 9:** Imaging entropies of three different algorithms.

	The 2D ISRJ Results	The Anti-Jamming Results without Eliminating Sidelobe	The Anti-Jamming Results in This Paper
Entropy	0.5508 dB	0.5448 dB	0.4260 dB
RMSE	27.8921	20.5304	1.3951

## Data Availability

Not applicable.
